# Cheap glass fiber mats as a matrix of gel polymer electrolytes for lithium ion batteries

**DOI:** 10.1038/srep03187

**Published:** 2013-11-12

**Authors:** Yusong Zhu, Faxing Wang, Lili Liu, Shiyin Xiao, Yaqiong Yang, Yuping Wu

**Affiliations:** 1 New Energy and Materials Laboratory (NEML), Department of Chemistry & Shanghai Key Laboratory of Molecular Catalysis and Innovative Materials, Fudan University, Shanghai 200433, China

## Abstract

Lithium ion batteries (LIBs) are going to play more important roles in electric vehicles and smart grids. The safety of the current LIBs of large capacity has been remaining a challenge due to the existence of large amounts of organic liquid electrolytes. Gel polymer electrolytes (GPEs) have been tried to replace the organic electrolyte to improve their safety. However, the application of GPEs is handicapped by their poor mechanical strength and high cost. Here, we report an economic gel-type composite membrane with high safety and good mechanical strength based on glass fiber mats, which are separator for lead-acid batteries. The gelled membrane exhibits high ionic conductivity (1.13 mS cm^−1^), high Li^+^ ion transference number (0.56) and wide electrochemical window. Its electrochemical performance is evaluated by LiFePO_4_ cathode with good cycling. The results show this gel-type composite membrane has great attraction to the large-capacity LIBs requiring high safety with low cost.

Greenhouse effect caused by gaseous emissions from the burning of fossil fuels and exhaustion of global energy resources urgently demand clean energy. Solar radiation, wind, and waves present green sources for energies that are variable in time, and energy storage systems of high power and energy densities are required. At the same time, the development of hybrid electric vehicles (HEVs), plug-in hybrid electric vehicles (PHEVs) and full electric vehicle (FEVs) also needs energy storage^1^,^2^,^3^,^4^,^5^. The great demands promote the needs for large-capacity lithium ion batteries (LIBs)^6^,^7^,^8^,^9^,^10^,^11^,^12^,^13^. Polymer electrolytes (PEs) provide a promising solution to replace the combustible organic liquid electrolytes leading to a marked improvement in safety for large capacity batteries^8^,^9^,^14^,^15^,^16^. Solid PEs still have quite some space because of the low ionic conductivity (10^−8^~10^−4^ S cm^−1^)^16^,^17^. Gel PEs, which are formed by absorbing a large amount of liquid electrolytes in polymer matrices and have characteristics of both the solid and the liquid electrolytes, become the optimal choice^18^. However, their poor mechanical property and high cost restrict their application^10^,^11^,^19^.

The separators for cheap lead-acid batteries, glass fiber mats (GFMs), which have the properties of low cost, good high-temperature stability and good resiliency against sustained pressure, are a nonwoven “paper” based on glass fibers manufactured by paper machines^20^. The porosity of the separator is about 90–95% range. However, their mechanical strength is poor. Their typical pore sizes vary from 1 to 100 μm with low uniformity. In contrast, lithium ion batteries require a separator with pore size in the range 0.03–0.1 μm, 30–60% porosity with high uniformity and acceptable mechanical strength^21^. As a result, GFMs could not be used for lithium ion batteries. However, low cost, high-temperature stability and simple manufacture process make glass fiber mats of great attractions to the lithium ion batteries.

By virtue of its attractive properties, poly(vinylidene fluoride) (PVDF) has been chosen as a polymer host for lithium ion batteries applications^22^. PVDF-based GPEs are highly electrochemically stable due to the presence of strong electron-withdrawing functional group (-C-F) and the polymer itself has a dielectric constant (*ε* = 8.4) which helps for greater dissolution of lithium salts and subsequently supports high concentration of charge carriers. Many systems were tested based on homo- and copolymers of PVDF activated by carbonates and lithium salts^23^,^24^,^25^. The conductivity values are generally high enough to envisage technological applications in battery systems. However, their mechanical strength remains still poor and cost still high.

Here we report a simple process to prepare a composite membrane, PVDF-GFM, based on PVDF and a GFM, which can be an excellent separator for lithium ion battery. It exhibits high safety (self-extinguishing) and its cost is much decreased in comparison with the commercial separator. Its ambient ionic conductivity is four times higher than that of commercial separator (Celgard 2730) after saturating with a LiPF_6_-containing electrolyte. The gelled composite membrane shows good electrochemical performance when evaluated by a commercial LiFePO_4_ cathode material.

## Results

**Figure 1:**
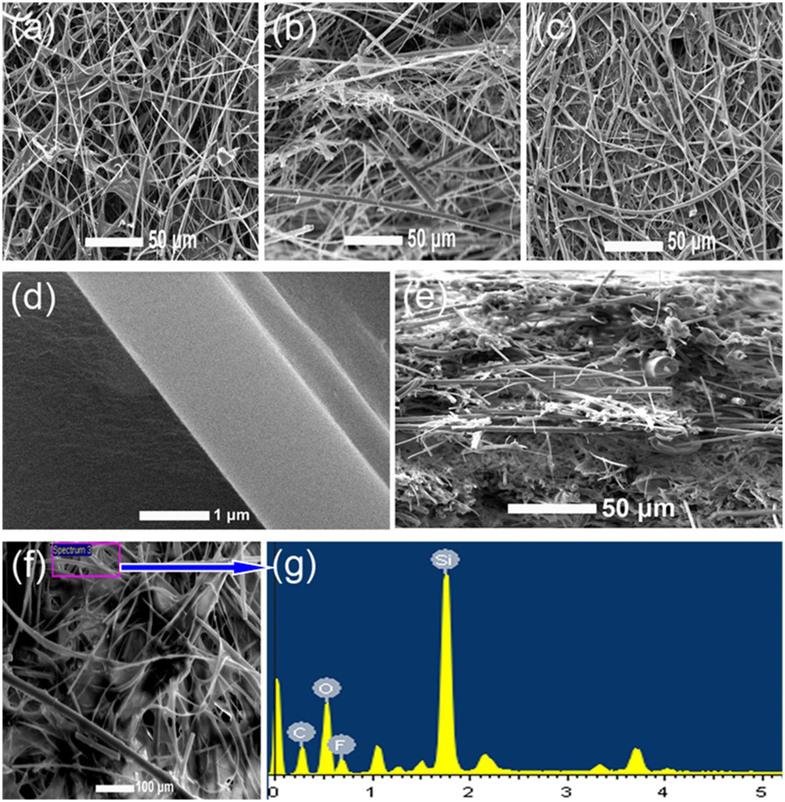
Physical characterization of the membranes. SEM micrographs for (a) the surface and (b) the cross-section of the GFM, (c, d) the surface and (e) the cross-section of the PVDF-GFM composite membrane, and (f, g) EDX of the PVDF-GFM membrane.

SEM micrographs of the GFM and the PVDF-GFM composite membrane are shown in Figure 1. From the surface and inner morphology of the GFM (Figure 1a and 1b), the pore size of the fibrous matrices is not uniform and the inner structure is very loose. In the case of the composite membrane, the pores of the fibrous matrix of the GFM are covered with PVDF and the inner space of the GFM membrane is also filled with PVDF matrix (Figure 1c and 1e). The pores on the surface of the composite membrane (Figure 1d) cannot be seen clearly. This indicates that the particles of electrodes in nano-size will not pass through the composite membrane while the Li^+^ ions can transport freely. This structure is of great importance to prevent possible micro short-circuit and help to improve the safety of LIBs, which is evidently advantageous over the commercial separator. The EDX analysis of the PVDF-GFM composite (Figure 1g) also shows that C, O, F and Si elements are observed on the inner GFM surface demonstrating that the GFM fibers are covered with PVDF.

**Figure 2:**
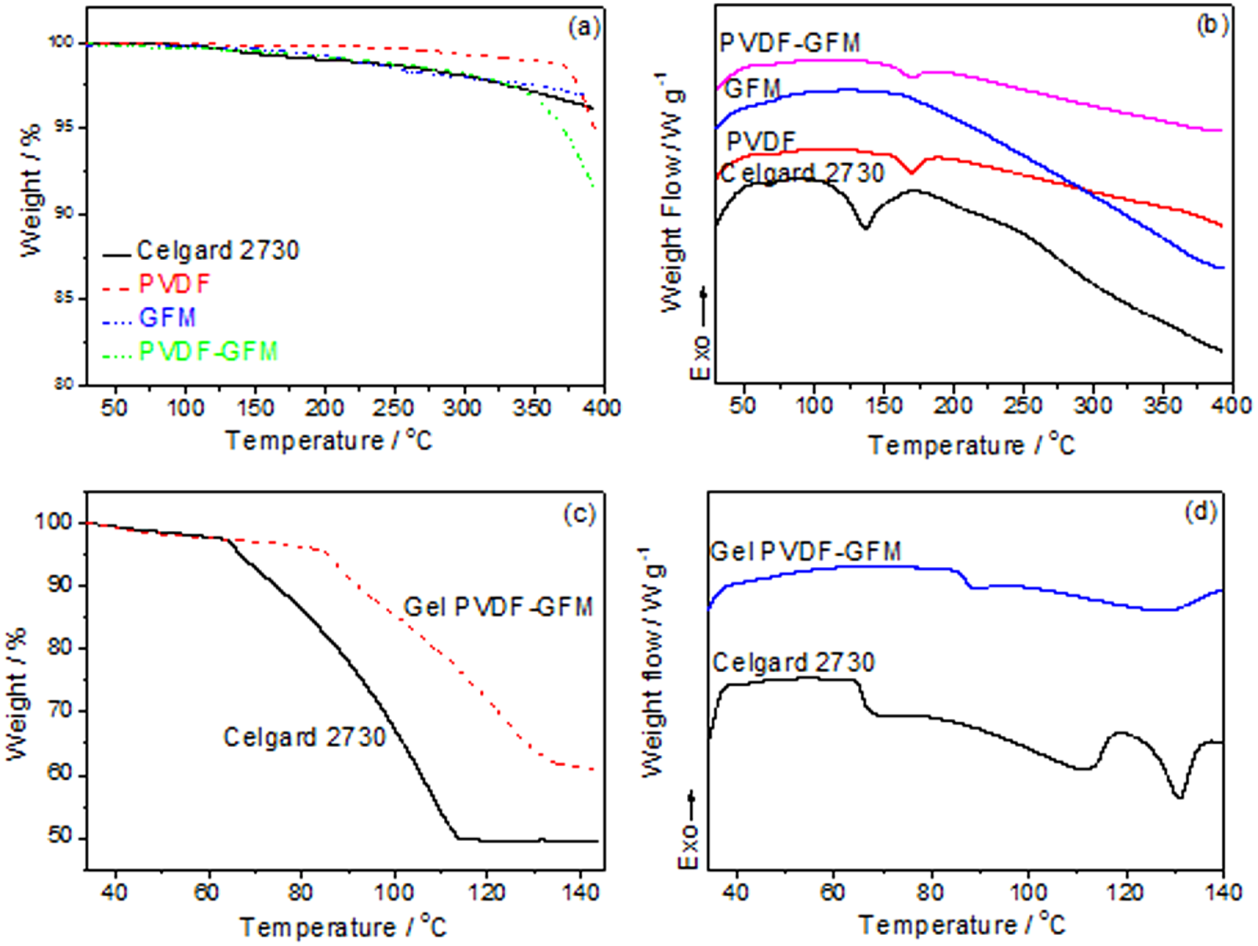
The thermal stability and the retention ability of the liquid electrolyte in the membranes. (a) Thermogravimetry (TG) and (b) differential scanning calorimetry (DSC) curves of the Celgard 2730, PVDF, the GFM and the PVDF-GFM composite membrane under air at the temperature rising rate of 10°C min^−1^, (c) TG and (d) DSC curves of the Celgard 2730 and the PVDF-GFM composite membrane after absorbed the same amount of LiPF_6_ electrolyte (1 ml g^−1^) at the temperature rising rate of 2°C min^−1^ under nitrogen.

Thermogravimetry (TG) and differential scanning calorimetry (DSC) curves of Celgard 2730 (a commercial separator), PVDF, the GFM and the PVDF-GFM composite membrane are shown in Figure 2a and 2b. The thermal stability of the PVDF-GFM composite membrane is about 170°C, which is 40°C higher than that of commercial separator, Celgard 2730. When the temperature is high above 170°C, the melt point of PVDF, the membrane does not appear clear sharp weight loss until 370°C, the same as PVDF.

The TG and DSC curves of the gel PVDF-GFM membrane and the Celgard 2730 separator soaked with the same amount of the electrolyte (1 mL g^−1^) are shown in Figure 2c and 2d. The organic electrolyte in the commercial separator starts to evaporate at 65°C which is indicated by the sharp endothermic peak in the DSC curve. In the TG curve it also presents a marked weight loss at the same temperature. When the temperature arrives at 112°C, the evaporable solvents disappear completely.

**Figure 3:**
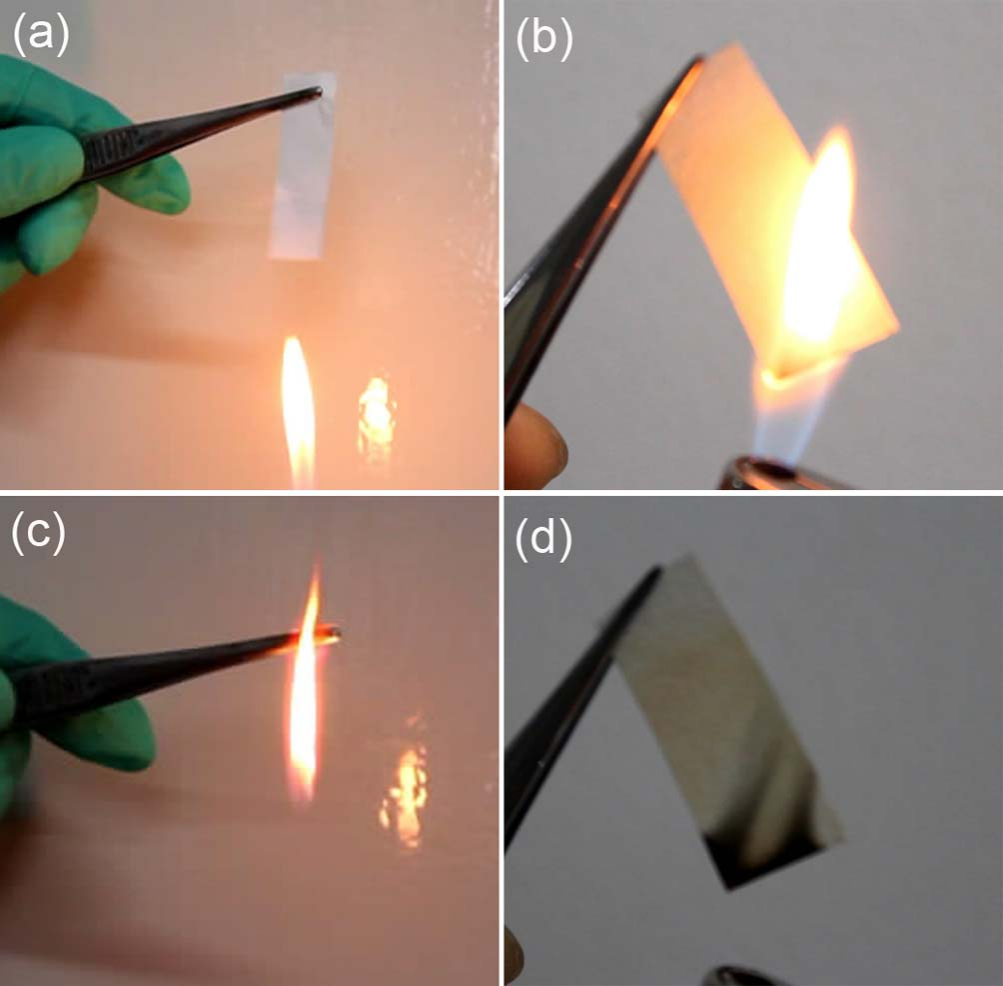
The flame retarding ability of the membranes. The combustion test of (a, c) the Celgard 2730 and (b, d) the PVDF-GFM composite membrane.

The combustion test of Celgard 2730 and the PVDF-GFM membrane is shown in Figure 3. When the commercial separator of Celgard 2730 was put on the fire, the separator shrank immediately and got on fire in a short time (< 3 s) (Figure 3c). This is similar to the other traditional separator due to the existence of combustible polyolefin matrix such polyethylene and polypropylene. The composite membrane shows perfect flame retarding ability. It did not catch on fire and also not shrink when putting on the fire (Figure 3d) because the components in the composite membrane are SiO_2_ and PVDF, which have flame retarding ability. The composite membrane shows acceptable mechanical property, which is very important for practical application of LIBs (Supplementary Fig. S1). The maximums of stress and strain of the PVDF-GFM composite are 14.3 MPa and 1.8%, respectively. After gelled to get GPE PVDF-GFM, the mechanical strength of the composite does not change.

**Figure 4:**
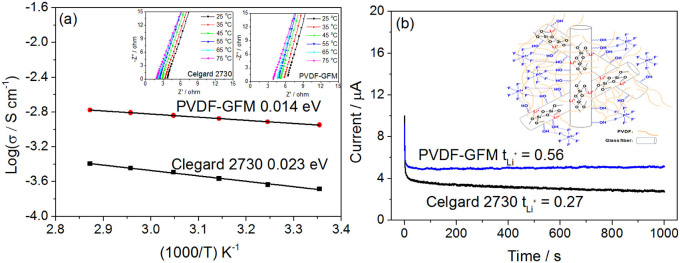
Lithium ion conductivity and transference number for the wetted membranes. (a) Impedance plots of the conductivity data at different temperatures and Arrhenius plots of the Celgard 2730 and the gel PVDF-GFM membrane; and (b) Chronoamperometry profiles for the Celgard 2730 and the gel PVDF-GFM membrane at 25°C in block cells using Li metal as both electrodes with step potential of 10 mV.

The uptake amount (the main factor for ionic conductivity) for the composite can be up to 132 wt.%, higher than that of Celgard 2730 (90.9 wt.%). This indicates that the ionic conductivity of the gelled composite will be at least at the same order of magnitude as that for the commercial separator. Figure 4a shows the ionic conductivities dependence on temperature for the commercial separator (Celgard 2730) and the gelled PVDF-GFM membrane at the range from 25 to 75°C. The conductivity was calculated from the impedance plots shown in the insets of Figure 4a. Typical impedance plots consist of a high frequency semicircle followed by a low frequency straight line, which correspond to contributions from the bulk/grain boundary and the electrode resistances, respectively. When the current carriers are ions and the total conductivity is the main result of ionic conduction, the plot shows the disappearance of the semicircular portion. The resistance of the bulk electrolyte has been retrieved from the intercept of the straight line on the real axis^27^. The ionic conductivity of the gel membrane at 25°C is 1.12 mS cm^−1^ and the value is five times that of the Celgard 2730 saturating with organic electrolyte (0.21 mS cm^−1^). This presents that the ionic conductivity of the GPEs is above the level for the commercial separator. The dependence of ionic conductivity on temperature can be reasonably fitted by the following equation (1):
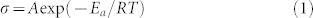
where *A* is the pre-exponential factor and *E_a_* is the activation energy. *E_a_* values are 0.014 eV and 0.023 eV for the PVDF-GFM gel electrolyte and Celgard 2730, respectively. That is, the movement of Li^+^ ions in the gel PVDF-GFM membrane is much easier than that in the Celgard 2730. The electrochemical stability of the gel PVDF-GFM electrolyte (Supplementary Fig. S2) is similar to that of the Celgard 2730, about 4.8 V, which is enough for LIBs. The transference numbers of Li^+^ ions are 0.27 and 0.54 for the Celgard 2730 and the gel PVDF-GFM membranes, respectively, which were estimated by chronoamperometry (Figure 4b) by comparing the initial and final current values.

**Figure 5:**
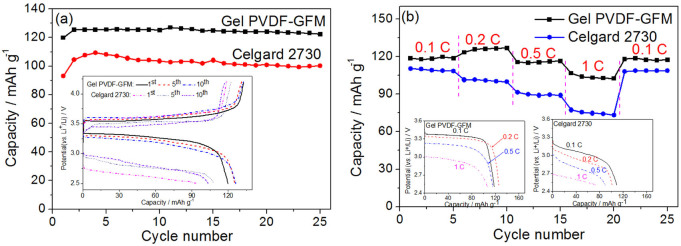
Electrochemical performance of the LiFePO_4_ cathode. (a) Cycling behavior and the charge-discharge curves at 1st, 5th and 10th cycles; and (b) rate behaviour and discharge curves at a charge current density of 0.2 C and discharge current density of 0.1 C, 0.2 C, 0.5 C, 1 C and 0.1 C, respectively. These were measured by using the Celgard 2730 or the PVDF-GFM composite membrane as separtors saturating with 1 mol L^−1^ LiPF_6_ electrolyte and Li metal as the counter and reference electrode.

The electrochemical performance of the gel PVDF-GFM membranes was evaluated by using LiFePO_4_ as the cathode and Li metal as the counter and reference electrode (Figure 5). The reversible capacity of LiFePO_4_ is about 125 mAh g^−1^ at 0.2 C for the gel membrane, which is higher than that for the commercial separator, about 100 mAh g^−1^ (Figure 5a). The cycling performance of the gel membrane is similar to that of the commercial separator. After 25 cycles there is still no evident capacity fading. From the corresponding charge-discharge curves, typical flat-shaped voltage profiles are observed around 3.2–3.5 V in the case of the gel membrane (Inset of Figure 5a), which are consistent with the reported coexistence reaction of two phases for the LiFePO_4_ cathode^29^,^30^,^31^, and the difference between charge and discharge curves is very small, less than 0.3 V. In the case of the Celgard 2730, the voltage profiles for the LiFePO_4_ are also flat (Inset of Figure 5a). However, the difference between charge and discharge voltages is larger, at least 0.8 V. Evidently, this higher voltage difference is due to the polarizations caused by the lower transference amount of Li^+^ ions. In addition, the gel membrane also presents satisfactory rate performance (Figure 5b) between 0.1 C and 1 C. When the composite was charged at 0.2 C and discharged at 0.1 C, 0.2 C, 0.5 C and 1 C, the capacity of the LiFePO_4_ tested with the gel PVDF-GFM is 118.7, 125.8, 115.3 and 103.1 mAh g^−1^, respectively, which is higher than that of the wetted Celgard 2730 (108.7, 100.5, 89.5, and 74.7 mAh g^−1^, respectively). When discharged with 0.1 C at last, the discharge capacity is recovered to the original value. Capacity retention and Coulombic efficience of the gel PVDF-GFM membrane in 40 cycles is very stable (Supplementary Fig. S3).

## Discussion

As the above shown, the GFM mainly consists of silicon dioxide which is thermally stable up to 400°C and the thermal stability of the composite membrane is mainly dependent on the PVDF. This means that even the polymer is melt down, the composite membrane can still keep the good shape up to 400°C, which is markedly higher than that for the commercial separator, less than 170°C. Even the temperature is up to 400°C, the direct contact of the two electrodes (the positive and negative ones) for LIBs is still impossible to lead to short circuit. It is also different from the ceramics coated polymers since the melt-down of the polymer host will lead to the movement of the ceramics and micro short-circuit can still easily happen when the temperature is above the melting point of the polymers^26^. This suggests that the mechanical thermal stability of our PVDF-GFM is superior to the present membranes or separator.

In the case of a lot of accidents such as fires and explosion of LIBs, the main reason is the emission of a large amount of combustible gases at the temperature below 120°C^6^. This means that the so-called shut-down behaivor of polyethylene in the commercial separator, which is at about 120°C, does not mean much for the safety improvement since LIBs usually do not work due to very low ionic conductivity at the temperature above 120°C. However, as to our gel PVDF-GFM membrane, as Figure 2 shows, the absorbed electrolyte begins to evaporate at 85°C which is 20°C higher than that of Celgard 2730, indicating the working temperature can be higher than that from the commercial separator. The main reason is that the organic electrolyte can be absorbed and retained by the PVDF matrix in the composite membrane. When the temperatur increases to 140°C, the gel PVDF-GFM shows only 35% weight loss. That is, a large proportion of electrolyte is still retained in the gel composite membrane, suggesting that LIBs can still work at 140°C. When the temperature is further increased, PVDF will melt down and present a different shut-down behavior, which will be of great value to improve the practical safety of lithium ion batteries. This suggests that our gel PVDF-GFM membrane can not only work at high temperature but also provide another shut-down behavior to improve the safety of LIBs.

As Figure 4 shows, the movement of Li^+^ ions in the gel PVDF-GFM membrane is much easier than that in the Celgard 2730. One reason is perhaps due to the polarity of the PVDF matrix, which hinders the movement of large PF_6_^−^ anions and assists the movement of Li^+^ ions^27^,^32^. Another reason can be ascribed to the surface of the GFM. These glass fibers, which are used for lead acid batteries, are hydrophilic, whose surfaces are easy to form hydrogen bond with fluorine atom in PF_6_^−^. The interaction hinders the movement of the anions of the electrolyte and improves the transference of lithium ions^28^, which is schematically illustrated in the inset of Figure 4b.

In our test, the LiFePO_4_ electrode pieces are a little thicker (17 mg cm^−2^, much larger than that of the most reports, about 2 mg cm^−2^). They need longer time to make the liquid electrolyte to disperse uniformly and finish the formation process. As a result, the discharge capacities in the initial several cycles are less than those in the successive cycles. After several cycles, the discharge capacity becomes stable. Since the LiFePO_4_ cathode is thicker, its voltage difference for the traditional separator during the charge and discharge process is also higher than the former reported^29^,^30^,^31^,^32^. However, due to the above higher ionic conductivity and transference number of Li^+^ ions in the gel PVDF-GFM membrane, LiFePO_4_ shows better electrochemical performance than that for the commercial separator.

In summary, after modification, cheap separator for lead-acid battery can be used in LIBs of high safety. The composite membrane exhibits high safety, good mechanical property and low cost. The ionic conductivity of the gelled membrane at ambient temperature is 4 times higher than that of the commercial separator (Celgard 2730) saturating with liquid electrolyte even the amounts of the liquid electrolyte are almost the same. Moreover, the lithium ion transference of the gel membrane at room temperature improves more than one time. The electrochemical performance of the gel membrane evaluated by using LiFePO_4_ as the working electrode and Li metal as the counter and reference electrode is very good, displaying higher discharge capacity and better rate capability in comparison with the commercial separator. These results suggest that this gel-type composite membrane is of great attraction to the LIB systems requiring high safety, better performance and low cost.

## Methods

### Preparation

PVDF (J & K Chemical, MW: ~540,000, 6 g) was dissolved into N, N-dimethylacetamide (70 mL, Sinopharm, AP) at 50°C for 2 h to give a clear solution. Cooled to room temperature, glass fiber mat (Changzhou Wujin Jinhui Battery separator factory, thickness: 175 μm) was fixed on a glass plate and cast-coated with the PVDF solution. After dried, another side was also coated by the same process to give the composite membrane, PVDF-GFM (thickness: 175 μm). The weight increases 58.7% after forming a composite with PVDF. The membrane was punched into circular pieces (*d* = 19 mm). Dried under vacuum at 80°C for 48 h, the pieces were soaked in a LiPF_6_ electrolyte (1 M LiPF_6_ in ethylene carbonate/dimethyl carbonate/ethyl methyl carbonate with weight ratio of 1/1/1) over 12 h in a glove box (water content: < 1 ppm) to get the gel polymer electrolyte (GPE) for further measurement.

### Characterization

Except stated, the following measurements were performed at room temperature. Thermogravimetric analysis (TGA) and differential scanning calorimetry (DSC) of the membranes were carried out by utilizing a Perkin-Elmer TGA7/DSC7. The surface morphology of the prepared membranes was investigated by Philips XL30 scan electron microscopy (SEM). The membranes were dipped into liquid nitrogen and broken into two parts, and then the SEM micrographs of the cross-sections were taken. All samples were sputtered with gold prior to the SEM measurement. Energy dispersive X-ray spectroscopy (EDX) analysis was conducted with the same SEM instrument to confirm the elemental composition of the sample. The thickness of the membranes was measured with a micrometer (SM&CTW, Shanghai). Stress-strain test was conducted using a Sansi YG832 tensile testing machine with a crosshead speed of 1 mm min^−1^. The width of the sample was 4 mm.

### Uptake ability

The amount of liquid electrolyte uptake (*η*) is calculated as the equation (2):
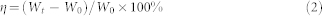
where *W_0_* and *W_t_* are the weights of the membranes before and after absorption of the organic electrolyte, respectively. The weight was weighed in a glove box.

### Ionic conductivities

The ionic conductivities were measured by electrochemical impedance spectroscopy (EIS). The samples were measured in blocking-type cells where the GPE membrane or the commercial separator saturated with the electrolyte was sandwiched between two stainless steel electrodes. Impedance data were obtained with an electrochemical working station CHI660C (Chenhua) in the frequency range 10 Hz – 100 kHz between 25 and 75°C. The ionic conductivity was calculated from the equation (3):
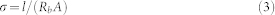
where *σ* is the ionic conductivity, *R_b_* is the bulk resistance, *l* is the thickness of the polymer electrolyte or separator and *A* is the area of the stainless steel electrode.

### Linear sweep voltammogram

The linear sweep voltammogram was obtained by the electrochemical working station CHI660C using a two-electrode cell. Stainless steel was used as the working electrode and lithium foil as the counter and reference electrode, respectively. The measurement was done between 0 and 6 V (vs. Li^+^/Li) at the scan rate of 2 mV s^−1^ at 25°C.

### Chronoamperometry

The chronoamperometry profile was obtained by the electrochemical working station CHI660C measuring in blocking-type cells where the GPE membranes were sandwiched between two lithium metal electrodes. The step potential was 10 mV. The lithium ion transference number was calculated according to the following equation:
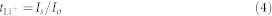
where *I*_s_ and *I*_o_ represent the currents at the steady state and initial state, respectively.

### Electrochemical performance

Test of electrochemical performance of the membranes was conducted by assembling 2025 coin cells with lithium metal foil as the counter and reference electrode. The working electrode was prepared by coating the N-methyle-2-pyrrolidone-based slurry containing LiFePO_4_ (STL Energy Technology Co., Ltd., China), acetylene black and PVDF in a weight ratio of 8:1:1 on aluminium foil (thickness: 20 μm) using the doctor-blade technique, and the cast foils were then punched into circular pieces (*d* = 15 mm) and dried at 120°C for 12 h under vacuum. The mass loading of LiFePO_4_ was around 17 mg cm^−2^. The GPE membrane was used as the separator and electrolyte. All cells were assembled in an Ar-filled glove box. Cycling test was carried out by a Land tester (CT2001A) between 2.5 and 4.2 V at a current density of 0.2 C (1C is 170 mA g^−1^) based on LiFePO_4_. Rate test was carried out by a Land tester (CT2001A) between 2.5 and 4.2 V based on LiFePO_4_ cathode at a charge current density 0.2 C and at a successive discharge current density of 0.1 C, 0.2 C, 0.5 C, 1 C and 0.1 C, respectively. Every discharge rate was tested for five cycles.

## Author Contributions

Y.W. proposed the conceptual idea, participated in the analysis of results, discussing and writing the manuscript, and provided financial support through grant application. Y.Z. prepared the composite membrane, tested its performance and participated in writing this manuscript. F.W. and L.L. prepared the cathode, S.X. and Y.Y. provided the data on control separator. All authors read and approved the final manuscript.

## Supplementary Material

Supplementary Information Supplementary Information
