# Supplementary material for: Cheap glass fiber mats as a matrix of gel polymer electrolytes for lithium ion batteries
Source: Sci Rep. 2013 Nov 12;3:3187. doi: 10.1038/srep03187 (PMC3824160; doi:10.1038/srep03187)
Supplement: Supplementary Information [file srep03187-s1.pdf]

Supplementary Information

Cheap Glass fiber mats as a matrix of gel polymer electrolytes for lithium ion batteries

Yusong Zhu, Faxing Wang, Lili Liu, Shiyin Xiao, Yaqiong Yang, Yuping Wu*

1. The mechanical property of the gel PVDF-GFM

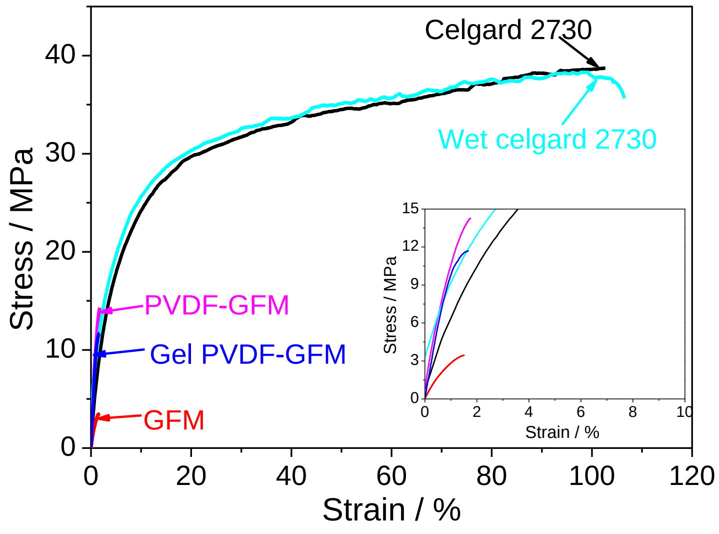

Figure S1 Stress–strain curves of the Celgard 2730, the wet Celgard 2730, the GFM, the PVDF-GFM composite membrane and the gel PVDF-GFM.

As shown in Figure S1, the maximums of stress and strain of the PVDF-GFM composite are 14.3 MPa and 1.8%, respectively, which are smaller than that of the Celgard 2730. In comparison with the pure GFM, it is much increased, indicating that the PVDF-GFM composite membrane is acceptable for the use of battery systems. The main reason is that the surface of the fibrous matrix of the GFM is covered with PVDF and some inner space of the GFM membrane is also filled with PVDF matrix which is helpful to improve the mechanical property of the PVDF-GFM composite. After gelation, the maximums of stress and strain of the gel PVDF-GFM become smaller than the dry state of PVDF-GFM. The reason is that the gelation of PVDF in the GFM changes the

mechanical property of the PVDF-GFM. The strength of the commercial separator is not influenced by the organic electrolyte.

2. The electrochemical window of the PVDF-GFM

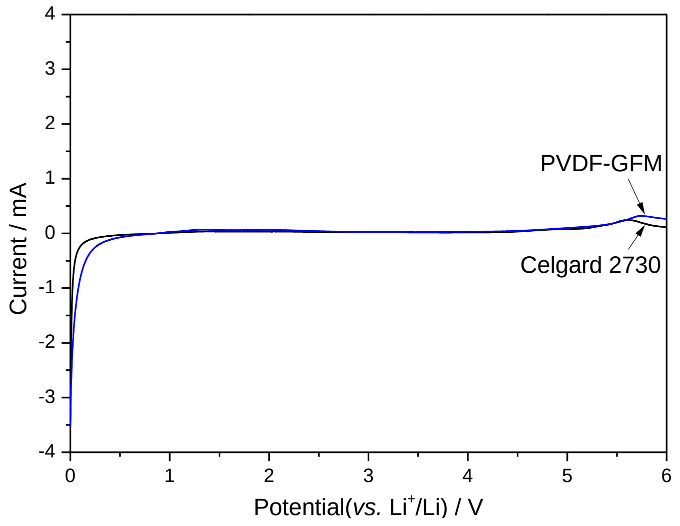

Figure S2 The linear sweep voltammograms of the Celgard 2730 and the PVDF-GFM membraneds after saturated by the $LiFP_6$ electrolytes.

As can be seen from Figure S2, no current peak is found through the working electrode from open circuit potential to 4.8 V (vs. Li^+/Li) for commercial separator Celgard 2730 saturating with the organic electrolytes. When the open circuit potential is more than 4.8 V, the $LiPF_6$ electrolytes begin to decompose and there is a current peak. The result is consistent with that for the commercial lithium ion batteries. In the case of the electrochemical stability of the gel PVDF-GFM composite membrane, it is similar to that of Celgard 2730 since the amount of the organic electrolyte is almost the same and contacts directly with the electrodes. That is, the electrochemical window of the gel PVDF-GFM membrane is also about 4.8 V, which is enough for lithium ion batteries.

3. Cycling and coulombic efficiency behavior of the $LiFePO_4$ cathode tested by the gel PVDF-GFM

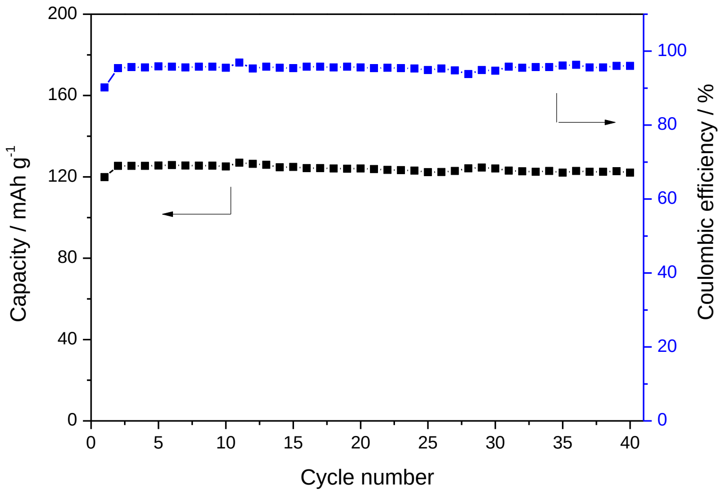

Figure S3 Cycling and coulombic efficiency behavior of the $LiFePO_4$ cathode tested by the PVDF-GFM composite membrane saturating with 1 mol L^{-1} $LiPF_6$ electrolyte as separtors and Li metal as the counter electrode.
